# Efficacy of low-intensity pulsed ultrasound treatment for surgically managed fresh diaphyseal fractures of the lower extremity: multi-center retrospective cohort study

**DOI:** 10.1007/s00776-013-0358-5

**Published:** 2013-03-06

**Authors:** Yo Kinami, Tomoyuki Noda, Toshifumi Ozaki

**Affiliations:** 1 Department of Community Medicine, Okayama University Graduate School of Medicine, Dentistry and Pharmaceutical Sciences, 2-5-1 Shikata-cho, Kita-ku, Okayama, Okayama 700-8558 Japan; 2 Department of Orthopedic Surgery, Okayama University Hospital, 2-5-1 Shikata-cho, Kita-ku, Okayama, Okayama 700-8558 Japan; 3 Department of Science of Functional Recovery and Reconstruction (Orthopedic Surgery), Okayama University Graduate School of Medicine, Dentistry and Pharmaceutical Sciences, 2-5-1 Shikata-cho, Kita-ku, Okayama, Okayama 700-8558 Japan

## Abstract

**Background:**

There are no evidence on the effects of low-intensity pulsed ultrasound (LIPUS) on surgically managed fresh fractures. We therefore performed a multicenter retrospective cohort study to investigate the effects of LIPUS on surgically managed fresh fractures.

**Methods:**

This study included patients surgically treated for diaphyseal fractures of the femur or tibia between August 2009 and July 2010 at 14 institutions. Outcome was the union period. We performed an overall comparison of the LIPUS group (78 cases) with the control group (63 cases), as well as subgroup analyses comparing outcomes for fracture sites, fracture types, soft tissue conditions, and fixation methods between the groups.

**Results:**

There was no significant difference between the groups in terms of distribution of cases by fracture site, fracture type, soft tissue condition, fixation method. Analyses comparing subgroups, however, showed significant differences between the two groups, particularly in relation to type C fractures, regardless of whether all cases or only closed-fracture cases were analyzed: there was an approximately 30 % reduction in the union period for type C fractures in the LIPUS group. There were also cases requiring reoperation due to lack of stability, even among the type C fractures.

**Conclusions:**

LIPUS is effective for surgically managed, fresh, type C comminuted diaphyseal fractures of the lower limbs when there is appropriate stability at the fracture site.

## Introduction

The conditions needed for ultrasound-promoted bone union in an animal fracture model were first published by Duarte in 1983 [1]. The effects of low-intensity pulsed ultrasound (LIPUS) on bone union in fracture repair were subsequently confirmed in a range of basic studies. The clinical effects of LIPUS on both conservatively treated fresh fractures and surgically managed fractures with delayed union or nonunion have since been confirmed.

In 1998, Japanese health insurance began to cover LIPUS as a treatment for delayed union and nonunion. In 2008, it was also made available for fresh, postoperative, open, or comminuted fractures. However, substantiated evidence for the effects of LIPUS on surgically managed fresh fractures is still lacking. Accordingly, we investigated the effects of LIPUS on this type of fracture by performing a multicenter retrospective cohort study at Okayama University, in collaboration with Okayama University’s associated hospitals.

## Materials and methods

We performed our study in collaboration with 14 hospitals associated with the author’s university, and divided the hospitals into those that actively use LIPUS (“active hospitals”) and those that do not use LIPUS (“nonactive hospitals”). We prospectively gathered information on cases involving the use of LIPUS from active hospitals according to the criteria and protocol given below. In addition, we based our control group on cases that did not involve the use of LIPUS, which we gathered retrospectively during the same period and according to the same criteria from both the active and nonactive hospitals. Subjects were patients who received surgery for diaphyseal fractures of the femur or tibia between August 2009 and July 2010. Patients in the LIPUS group received therapy with the SAFHS2000J (Teijin, Tokyo, Japan). We used the same follow-up protocol for both control and LIPUS cases. Approval was obtained from the institutional review board, and informed consent was provided by all subjects. Differences between groups were analyzed using the Mann–Whitney *U* test and Pearson’s chi-square test where appropriate. Results were considered statistically significant when *P* <0.05.

### Criteria and protocol

Criteria for inclusion in the study were: ≥16 years of age, spoke Japanese, consented to participate in the follow-up, and had a fresh femoral or tibial diaphyseal fracture—either open (Gustilo type I, II, or IIIa) [2, 3] or closed—for which LIPUS was available within 3 weeks of injury. We excluded patients who were <16 years of age, had fractures in bones other than the femur or tibia, had a metaphyseal or pathological fracture or a refracture, had a Gustilo type IIIb or IIIc open fracture or periprosthetic fracture, or did not consent to participate in the follow-up.

Outcome was the time until union (union period). Two experimental orthopedic surgeons, the attending surgeons, and the surgeons from the other hospitals associated with our university determined the point of bone union, defined as the point when cortical bony continuity was found at three sites or more using bidirectional X-rays, while also taking into consideration the clinical findings and course. We considered bony continuity to be the point at which the callus matured. Follow-up consisted of monthly radiography until union was confirmed and then follow-up surveys until rehabilitation was confirmed. We performed LIPUS for at least 3 months until union was achieved. Although treatment during recovery was subject to the protocol of each hospital, partial weight bearing began from an average of 1 month postoperatively, with subsequent progression to full weight bearing dependent on the level of callus formation.

## Results

**Fig. 1:**
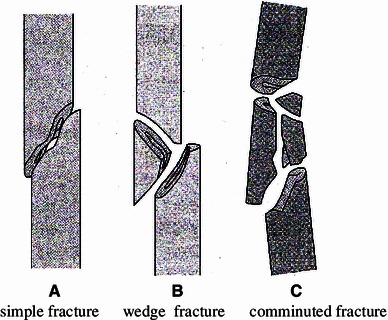
AO/ASIF comprehensive classification of fractures

Ninety cases were registered in the LIPUS group, but 12 were excluded because of a lack of adequate follow-up data. There were 88 cases from the same period in the control group, with 25 excluded because of a lack of proper follow-up. We had 78 cases in the LIPUS group (51 males and 27 females, mean age 48.7 years) and 63 cases in the control group (38 males and 25 females, mean age 46.9 years). In the LIPUS group, therapy was started within 3 weeks after the injury. We found no significant difference between the groups in terms of the distribution of cases by fracture site, fracture type (AO classification A/B/C) [4] (Fig. 1), soft tissue condition, and fixation. With regard to final outcome, there were four cases requiring revision surgery in the LIPUS group and one in the control group (Table 1).

**Table 1:** Baseline characteristics

	Control (*n* = 63)	LIPUS (*n* = 78)	Total (*n* = 141)	*P* value
Gender				
Male	38	51 (4^a^)	89	0.8
Female	25 (1^a^)	27	52	
Age	46.9 years old (range 16–94)	48.7 years old (range 16–95)	141	0.64
Fracture site				
Femur	29 (1)	37 (2)	66	0.81
Tibia	34	41 (2)	75	
AO classification				
A	15	15 (1)	30	0.53
B	32 (1)	35	67	
C	16	28 (3)	44	
Soft tissue				
Open	21	25 (3)	46	0.64
Closed	42 (1)	53 (1)	95	
Surgery				
Nail	57 (1)	67 (4)	124	0.42
Plate	6	11	17	
Result				
Union	62	74	136	0.25
Nonunion (reoperation)	1	4	5	

^a^Number of cases requiring revision surgery

**Fig. 2:**
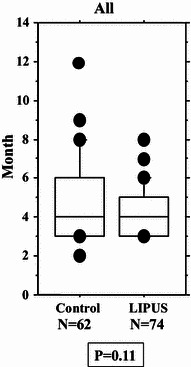
No significant difference in union period was found between the two groups overall (i.e., including all cases)

**Fig. 3:**
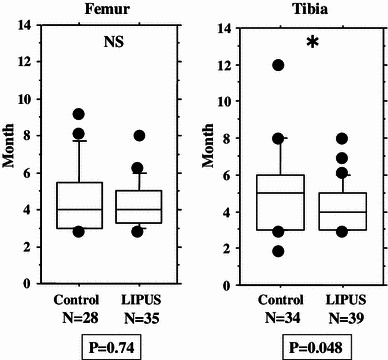
No significant difference between the two groups in union period was found when the fracture site was the femur, but a significant difference was found for the tibia

**Fig. 4:**
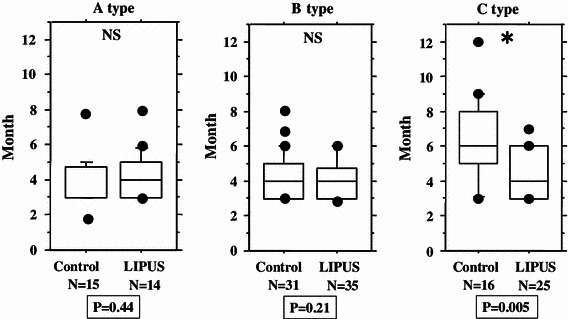
No significant difference between the two groups in union period was found when the fracture type was A, or when it was B, but a significant difference was found for type C fractures

**Fig. 5:**
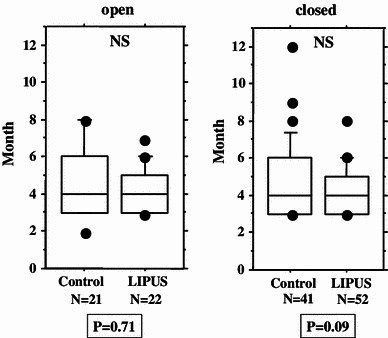
No significant difference between the two groups in union period was found for open fractures only or for closed fractures only

**Fig. 6:**
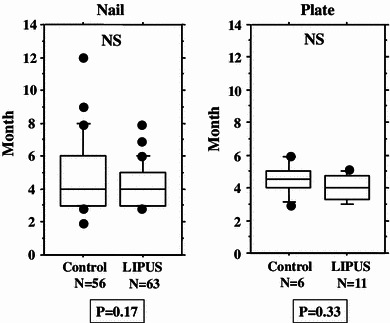
No significant difference between the two groups in union period was found for nailed fractures only or for fractures treated with plate fixation only

**Fig. 7:**
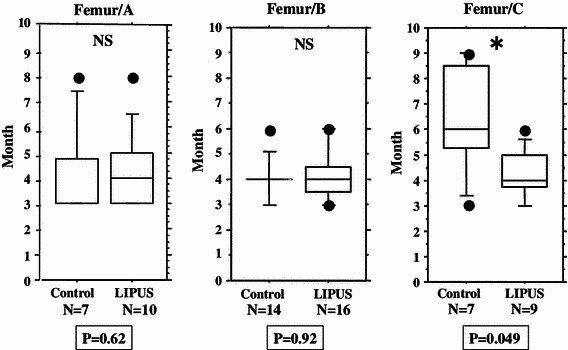
No significant difference between the two groups in union period was found for femur/type A fractures only and femur/type B fractures only, but there was a significant difference for femur/type C fractures

**Fig. 8:**
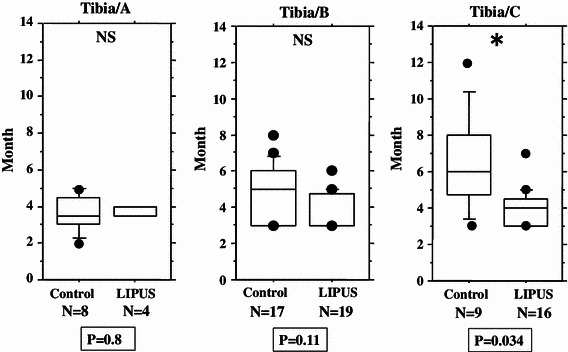
No significant difference between the two groups in union period was found for tibia/type A fractures only and tibia/type B fractures only, but there was a significant difference for tibia/type C fractures

**Fig. 9:**
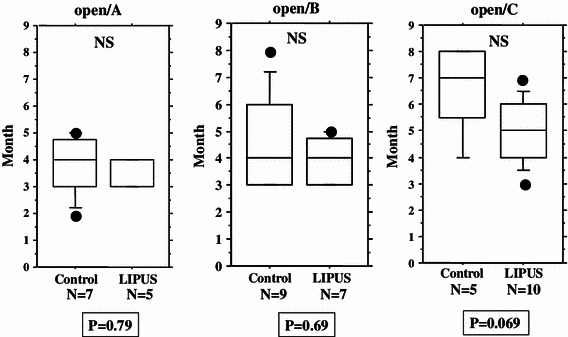
No significant difference between the two groups in union period was found for open/type A fractures only, open/type B fractures only, or open/type C fractures only

**Fig. 10:**
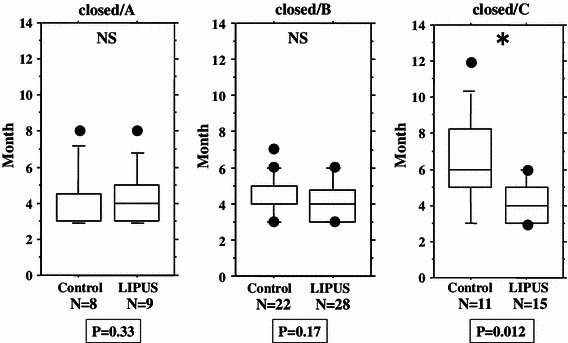
No significant difference between the two groups in union period was found for closed/type A fractures only and closed/type B fractures only, but there was a significant difference for closed/type C fractures

We performed an overall statistical analysis as well as subgroup analyses of union period by fracture site, fracture type, soft tissue condition, and fixation for both the LIPUS and control groups. The overall comparison of the groups did not find any significant differences, with overall mean union periods of 4.2 and 4.8 months observed for the LIPUS and control groups, respectively. A subgroup analysis of fracture site showed a significant difference between the groups for “tibia,” and a subgroup analysis of fracture type found a significant difference between the groups for “type C” fractures (Table 2; Figs. 2, 3, 4, 5, 6). We performed additional subgroup analyses of the combinations “fracture site/fracture type” and “soft tissue condition/fracture type” because of the marked difference in outcome for type C fractures between the groups. These analyses showed significant differences in outcome for femur/type C, tibia/type C, and closed/type C fractures, with the union period being approximately 30 % shorter in the LIPUS group for these fracture site/type combinations (Table 3; Figs. 7, 8, 9, 10).

**Table 2:** Subanalysis of outcome (mean union period in months) focusing on fracture site and type, soft tissue condition, and type of surgery for both groups

	Control	LIPUS	*P* value
All	4.8	4.2	0.11
Fracture site			
Femur	4.7	4.3	0.74
Tibia	4.9	4	0.048
AO classification			
A type	3.9	4.1	0.44
B type	4.9	4	0.21
C type	6.4	4.5	0.005
Soft tissue			
Open	4.7	4.3	0.71
Closed	4.8	4.1	0.09
Surgery			
Nail	4.8	4.2	0.17
Plate	4.5	4	0.33

Outcomes for the underlined fracture site and type were found to differ significantly between the groups (*P* value <0.05)

**Table 3:** Subanalysis of outcome (mean union period in months) focusing on specific combinations of fracture site and type for both groups

	Control	LIPUS	*P* value
Femur/A	4.1	4.4	0.62
Femur/B	4.1	4.0	0.92
Femur/C	6.4	4.2	0.049
Tibia/A	3.6	4.0	0.8
Tibia/B	4.6	4.4	0.11
Tibia/C	6.4	4.0	0.034
Open/A	3.7	3.6	0.79
Open/B	4.4	3.8	0.69
Open/C	6.6	5.0	0.069
Closed/A	4.0	4.4	0.33
Closed/B	4.4	4.0	0.17
Closed/C	6.4	4.2	0.012

Outcomes for the underlined fracture site/type combinations were found to differ significantly between the groups (*P* value <0.05)

Although the reoperation rate was high in the LIPUS group, there was no statistically significant difference in reoperation rate between the two groups. We analyzed the five cases that required revision surgery. One case originally had a large (5 cm) bony defect, but union was achieved outside of the defect area. In the other four cases, the smaller nails used in the first operation were insufficient, resulting in nonunion due to a lack of stability. Bone union was achieved with a bone graft in the first case and via exchange nailing [5, 6] in the other four cases (Table 4).

**Table 4:** Cases requiring revision surgery

Group	Age	Gender	Site	Classification	Soft tissue	Surgery	Revision surgery	Comments
LIPUS	18	Male	Tibia	C	Open III A	Nail	Bone graft	Large bone defect
LIPUS	65	Male	Tibia	C	Open II	Nail	Exchange nail	Unreamed nail
LIPUS	37	Male	Femur	C	Open I	Nail	Exchange nail	Unreamed nail
LIPUS	18	Male	Femur	A	Close	Nail	Exchange nail	Distal diaphyseal fracture
Control	19	Female	Femur	B	Close	Nail	Exchange nail	Gap 5 mm

5 cases had revision surgery. 1 case received a bone graft because of the large bone defect; the other 4 cases underwent exchange nailing because of nonunion

All cases ultimately achieved union

**Fig. 11:**
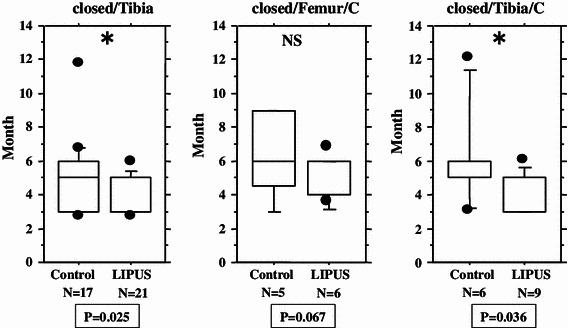
Significant differences between the groups in union period were found for tibial fractures only and tibia/type C fractures only. Femur/type C fractures in the LIPUS group tended to have a shorter union period than those in the control group

We performed separate subgroup analyses of the open and closed fracture groups. However, we could not perform reliable statistical analysis on the open fracture group (LIPUS 21 cases, control 22 cases) because there were too few cases and large differences. On the other hand, the closed fracture group (LIPUS 53 cases, control 42 cases) was analyzed using the same method as employed for the overall analysis (Tables 5, 6), although the number of cases with closed type A tibial fractures was too small, so this combination could not be analyzed statistically. The closed fracture group analysis showed significant differences in outcome between the groups for type C fractures, tibial fractures, and tibia/type C fractures, and there was a tendency (*P* = 0.067) for the union period to be shorter in the LIPUS group than in the control group for femur/type C fractures (Figs. 10, 11).

**Table 5:** Analysis of baseline characteristics for the closed fracture group

	Control (*n* = 42)	LIPUS (*n* = 53)	Total (*n* = 95)	*P* value
Gender				
Male	22	32 (1^a^)	54	0.43
Female	20 (1^a^)	21	41	
Age	47.7 years old (range 16–94)	50.0 years old (range 17–95)	95	0.65
Fracture site				
Femur	25 (1)	32 (1)	57	0.93
Tibia	17	21	38	
AO classification				
A	8	10 (1)	18	0.99
B	23 (1)	28	51	
C	11	15	36	
Surgery				
Nail	37 (1)	45 (1)	82	0.65
Plate	5	8	13	
Result				
Union	41	52	136	0.87
Nonunion (reoperation)	1	1	5	

^a^ Number of cases requiring revision surgery

**Table 6:** Subanalysis of outcome (mean union period in months) focusing on fracture site and type (and combinations thereof) for closed fractures in both groups

	Control	LIPUS	*P* value
Fracture site			
Femur	4.6	4.3	0.84
Tibia	5.1	3.8	0.025
AO classification			
A type	4.0	4.4	0.33
B type	4.4	4	0.2
C type	6.4	4.1	0.018
Surgery			
Nail	4.9	4.2	0.2
Plate	4.6	3.8	0.14
Femur/A	4.1	4.4	0.46
Femur/B	4.2	4.2	0.86
Femur/C	6.4	4.5	0.067
Tibia/A	–	–	–
Tibia/B	4.6	3.8	0.12
Tibia/C	6.3	3.9	0.036

Outcomes for the underlined fracture site, type, and site/type combination were found to differ significantly between the closed fracture subgroups of the LIPS and control groups (*P* value <0.05)

## Discussion

Basic research, including in vitro [7] and animal studies [8–10], has shown that LIPUS accelerates the repair reactions involved in bone union at the cellular level. Busse [11] published a systematic review of clinical studies on LIPUS therapy in 2009. In conservatively treated fresh fractures, an analysis of 67 cases of diaphyseal fracture of the tibia [12], 61 cases of distal radius fracture [13], and 30 cases of scaphoid fracture [14] found LIPUS therapy to be effective. Union periods in the LIPUS group were 30–40 % shorter than those in the control group. Multicenter analysis [15, 16] also showed that LIPUS is effective in cases of delayed union and nonunion. The effects of LIPUS in surgically treated fresh fractures are not, however, as clear. One study demonstrated no effect of LIPUS in 32 cases of diaphyseal fracture of the tibia with intramedullary nailing [17]. Another study showed that LIPUS shortened the period of cortical bridging, with callus formation in 11 cases of diaphyseal fracture of the tibia with intramedullary nailing and in 19 cases with external fixation [18]. However, we did not find any other studies demonstrating the effects of LIPUS on surgically managed fresh fractures.

Although there was no significant difference in the union period between the LIPUS and control groups overall (i.e., for all cases) in this study, we found significant differences between these groups when we performed subgroup analyses—mainly when we focused on type C fractures. It is difficult to precisely irradiate the fracture site in the femur, as the irradiation site is not easy to determine [19]. However, the target for irradiation in a type C fracture, with its wide fracture area, is larger than the targets in type A and B fractures, so targeting is easier for type C fractures, even in the femur. In addition, early weight bearing is possible in types A and B if the main segments are stabilized by bony contact after fixation. In these fracture types, stimulation through early and appropriate weight bearing [20] may already lead to the maximum potential for union at the fracture site, in which case LIPUS would have no additional stimulatory effect on the fracture. On the other hand, the fact that there was a significant difference between the two groups for type C fractures (in which contact cannot be achieved between the main fragments) suggests that the stimulatory effect of LIPUS is equivalent to that of an appropriate weight-bearing stimulus. LIPUS may be particularly useful for this fracture type, because type C fractures cannot tolerate early weight-bearing stimulus between the segments, even with surgery.

Soft tissue condition was found to influence the union period. However, when we focused on each soft-tissue condition, we did not find a significant difference in outcome between the groups, except for closed type C fractures (Figs. 5, 9, 10). Because Gustilo type IIIb and IIIc fractures were excluded from this study, there is the possibility that the vascular conditions around the fracture site in open fractures were almost the same as those in closed fractures. This may have led to almost the same outcome results in the analysis of all cases as in the analysis of the closed fractures only.

Since the treatment was administered by a variety of surgeons in this multi-center study, we focused on diaphyseal fractures of the femur and tibia in order to minimize bias due to differences in surgical technique, because the operative method was standardized for these fracture sites and because determination of bone union is easier at these sites than at others. We attempted to minimize the bias still further by ensuring that the surgeons were the members of the trauma group of our university, that they had been licensed for over 10 years, that each was the chief director of the orthopedic trauma service at one of the hospitals included in the study, and that they had completed the AOTrauma advance course. 

The assignment of cases could not be randomized in this study design. Because LIPUS treatment for fresh postoperative fractures is covered by Japanese health insurance, it would have been ethically problematic to establish a control group, so we were compelled to perform a retrospective cohort study. However, we consider this study to have the same significance as a prospective case series investigating the effects of LIPUS therapy on fresh fractures.

LIPUS does not compensate for a lack of stability. Thus, even in type C fractures (for which LIPUS was found to be effective in this study), revision surgery was still necessary when the fracture was unstable. For any fracture that may lack stability and in which union has still not occurred 3 months postoperatively, reoperation (changing the fixation or adding a bone graft) should be considered instead of continuing LIPUS, which is ineffective in this situation.

## Conclusion

We investigated the effect of LIPUS on surgically managed fresh fracture cases involving the shaft of the femur or tibia by performing a multi-center retrospective cohort study. We analyzed the outcomes (i.e., union periods) of 78 cases in our LIPUS group and 63 cases in our control group. Although there was no significant difference in outcome between the two groups overall, LIPUS appeared to be highly effective, with significant differences observed in a subgroup analysis of type C fractures in particular; for type C fractures, LIPUS facilitated an approximately 30 % decrease in the union period. However, there were cases requiring revision surgery due to a lack of stability, even among the type C fractures. Therefore, LIPUS is effective for type C fractures that are sufficiently stable at the fracture site.
